# Long COVID sequelae in heart transplant recipients

**DOI:** 10.1016/j.jhlto.2026.100600

**Published:** 2026-05-26

**Authors:** Christopher R. Lees, Tyler Morad, Martine Webb, Myung S. Sim, Jeffrey J. Hsu

**Affiliations:** a Division of Cardiology, Department of Medicine, University of California Los Angeles, Los Angeles, California; b Division of Cardiology, Greater Los Angeles VA Medical Center, Los Angeles, California; c Department of Medicine, University of California Los Angeles, Los Angeles, California; d Cardiovascular Division, Department of Medicine, University of California Irvine, Irvine, California; e Department of Medicine Statistics Core, University of California Los Angeles, Los Angeles, California

**Keywords:** long COVID, heart transplant, COVID-19, post-acute sequelae of COVID-19

## Abstract

Post acute sequelae of SARS-CoV-2 infection (also known as Long COVID) have been defined as symptoms that persist after 3 months from initial acute episode of COVID-19. While the pathophysiology and mechanisms that cause Long COVID are hypothesized as secondary to persistent inflammation and immune dysregulation, the burden of this disease remains difficult to estimate due to varied symptomatology. Solid-organ transplant recipients in particular remain a vulnerable population that has been disproportionately affected by the COVID-19 pandemic, and the impact of Long COVID among orthotopic heart transplant recipients (OHTRs)—a patient population on chronic immunosuppressive therapy—remains incompletely characterized. We sought to evaluate patient-reported Long COVID symptoms between OHTRs compared to patients with baseline cardiomyopathy. We conducted a prospective survey-based study of adult OHTRs and cardiomyopathy control patients with documented SARS-CoV-2 infection. Participants completed telephone surveys, which included questionnaires of symptoms, quality of life (QOL), and mental health assessment. Between-group differences were evaluated using multivariable models adjusting for baseline characteristics. Our results show that the prevalence of Long COVID symptoms did not significantly differ between OHTRs and patients with cardiomyopathy. Dyspnea was reported more frequently in the non-OHTR group; however, this association was attenuated after adjusting for a higher burden of underlying pulmonary disease. No evidence of increased overall symptom burden was observed among OHTRs. In this first comparative analysis of Long COVID among OHTRs and control cardiomyopathy patients, OHTRs demonstrated no increase in symptom burden and may experience fewer persistent symptoms.

The COVID-19 pandemic has resulted in nearly 800 million confirmed cases and over 7 million deaths globally since its emergence in late 2019.^1^ While the acute phase of COVID-19 has been extensively studied, a growing public health concern has emerged in the form of post-acute sequelae of SARS-CoV-2 infection (PASC), commonly referred to as “Long COVID.”^2^, ^3^ This condition has been estimated to affect up to 10% to 30% of COVID-19 survivors and is characterized by persistent, non-specific symptoms involving multiple organ systems, most commonly: persistent fatigue, post-exertional malaise, shortness of breath, cognitive dysfunction, or problems with taste or smell.^4^, ^5^

Despite its increasing prevalence, the clinical manifestations and mechanisms of Long COVID remain poorly defined. Hypotheses for the mechanisms of Long COVID include persistent viral reservoirs, immune dysregulation, and autonomic dysfunction; however, more precise mechanistic pathways remain to be determined.^6^ The epidemiology of COVID-19 and its sequelae become even more complex when investigating its effects on vulnerable populations, including solid organ transplant recipients (SOTR). Between 2020 and 2022, SOTRs experienced a 7-fold higher risk of mortality from acute COVID-19 infection than the general US population, with COVID-19 accounting for more than one-fifth of all deaths in this group.^7^ While survival of acute COVID-19 infection is improving with vaccinations and established therapeutics, the long-term impact of COVID-19 in this specific population is far less understood.

Orthotopic heart transplant recipients (OHTR) represent one such uniquely vulnerable population where the pre-existing immunosuppression and common post-transplant comorbidities add layers of complexity to the myriad cardiac complications of COVID-19, including viral myocarditis, systemic inflammation, and endothelial dysfunction. Additionally, overlapping hallmark features of Long COVID, such as fatigue or dyspnea, are also common in OHTRs due to factors such as anemia, cardiac deconditioning, and medication side effects, further complicating efforts to distinguish the effects of Long COVID in this population.

Due to the growing prevalence of Long COVID and limited studies on the persistent effects of COVID-19 in heart transplant recipients, there is a need for targeted research in this specific patient population. The primary aim of this study is to evaluate the long-term sequelae of COVID-19 infection on patients with heart transplants from the perspective of symptom burden and overall quality of life. Given the concern for increased complications in this chronically immunosuppressed population, we hypothesized that OHTRs would have increased complications—including increased Long COVID symptomatology—compared with a control population.

## Methods

### Setting and study population

This study was reviewed and approved by the UCLA Institutional Review Board (Protocol #23–0122). Eligible participants for the study arm were identified through chart data abstraction of patients from UCLA Health from March 2020 to August 2023 who had (1) a diagnosis of a history of orthotopic heart transplantation and (2) a diagnosis of prior COVID-19 greater than 3 months prior to enrollment. For the control arm, individuals were included if they had (1) a diagnosis of cardiomyopathy (CMY) and (2) a diagnosis of prior COVID-19 greater than 3 months prior to enrollment. All subjects were aged 18 years or older.

Participants excluded from our study included patients undergoing active chemotherapy for malignancies or individuals who have participated in COVID-19 studies that involved therapies to lessen the impact of Long COVID.

Our initial study aim was intended to study 100 patients with OHT matched by age, sex, and race/ethnicity with 200 patients with cardiomyopathy (1:2 ratio) and without solid-organ transplantation. After recruitment and consenting, 55 study arm participants and 66 control arm participants were interviewed and included in the study. Due to recruitment limitations, matching was not performed, and analyses were adjusted for baseline differences using multivariable regression.

### Demographics

Demographic characteristics (age, sex, race, ethnicity) were obtained from the Electronic Health Record (EHR), as were medical history of hypertension, autoimmune disease, smoking history, chronic kidney disease, pulmonary disease, mental health disorders, and sleep apnea (Table 1). Baseline left ventricular ejection fraction (LVEF) as well as time elapsed between initial COVID-19 infection and enrollment were also gathered from the EHR. Race and ethnicity were self-identified. Similarly collected data included vaccination status at time of initial infection, level of medical care required for COVID-19 illness (hospitalization, intensive care unit stay, hospitalization greater than 14 days), use of non-invasive ventilation (NIV) (Bilevel Positive Airway Pressure [BiPAP] or high-flow nasal cannula [HFNC]), mechanical ventilation, and use of therapeutics for COVID-19 (Remdesivir, Dexamethasone, monoclonal antibodies [mAb], convalescent plasma, baricitinib, tocilizumab, nirmatrelvir-ritonavir). Lastly, the presence of immunosuppressive medications on patients’ medication lists was documented, including mycophenolate mofetil, calcineurin inhibitors, mTOR inhibitors, steroids, and azathioprine.

**Table 1:** Baseline Demographic and Clinical Characteristics

	**Controls (n = 66)**	**Cases (n = 55)**	***p*-value**	**Fisher's Exact Test**
**Age - y**	59.5	57.7	0.509	
**LVEF - %**	52.5	62.2	**<.001**	
**Time from 1**^**st**^**COVID infection - days**	735.7	707.6	0.399	
**Sex - no. (%)**				
Male	48 (72.7)	48 (87.3)	**0.049**	
Female	18 (27.3)	7 (12.7)		
**Hospitalization Data - no./total no. (%)**				
Hospitalized	12 (18.2)	29 (52.7)	**<0.001**	
Hospitalization >14 days	2 (3.03)	1 (1.82)	0.669	1
Admitted to intensive care unit	0 (0)	1 (1.82)	0.271	0.455
Use of non-invasive ventilation	1 (1.52)	3 (3.64)	0.455	0.59
**COVID Treatments Received - no./total no. (%)**				
Full Dose Vaccinations (≥2)	48 (72.7)	44 (80.0)	0.351	
Remdesivir	6 (9.09)	29 (52.7)	**<.001**	
Dexamethasone (or steroid equivalent)	4 (6.06)	7 (12.7)	0.204	
monoclonal antibody	2 (3.03)	6 (10.9)	0.082	0.139
Convalescent Plasma	0 (0)	12 (21.8)	**<.001**	
Baricitinib	1 (1.52)	0 (0)	0.359	1
Nirmatrelvir-Ritonavir)	20 (30.3)	0 (0)	**<.001**	<.001
**Past Medical History - no./total no. (%)**				
Autoimmune Disease	15 (22.7)	12 (21.8)	0.905	1
Smoking History	16 (24.2)	17 (30.9)	0.412	
Diabetes	27 (40.9)	26 (47.3)	0.482	
Hypertension	37 (56.1)	38 (69.1)	0.142	
CKD	18 (27.3)	17 (30.9)	0.661	
Pulmonary Disease	17 (25.8)	2 (5.45)	**0.003**	
Mental Health Disorders	22 (33.3)	15 (27.3)	0.471	
Sleep Apnea	17 (25.8)	6 (10.91)	**0.038**	
**Immunosuppression Agents- no./total no. (%)**				
Mycophenolate	1 (1.52)	34 (61.8)	**<0.001**	<.001
Calcineurin Inhibitors	0 (0)	54 (98.2)	**<0.001**	<.001
mTOR inhibitors	0 (0)	23 (41.8)	**<0.001**	<.001

Control patients (n = 66) were those who have a diagnosis of cardiomyopathy and COVID-19 infection without solid organ transplantation, and cases (n = 55) were patients who were OHT recipients and previous diagnosis of COVID-19.

### Telephone interviews

Participants were contacted by telephone and provided information regarding the study and then screened for their interest in participation. If the patient was willing to participate in the study, they were asked to complete an electronic consent. Interviewers would then conduct the interview using a scripted patient survey assessing their symptom burden related to Long COVID.

Our survey was composed of 3 main portions. The first portion of the survey consisted of a subjective assessment of 20 symptoms related to Long COVID. Participants were asked if they were currently experiencing more and/or worse symptoms at the time of interview specifically compared to before their COVID-19 infection. Symptoms included fatigue or tiredness after exertion, shortness of breath, loss or distorted sense of smell, loss or distorted sense of taste, heartburn/acid reflux, difficulty concentrating, worsened memory, insomnia, headaches, muscle/joint pain, chest pain, palpitations, chronic cough, dizziness/lightheadedness, tinnitus, changes in appetite, congestion, nausea/vomiting, visual disturbances, or peripheral neuropathy. These symptoms were scored as binary (yes or no), with a summative symptom burden scored for comparison.

The second portion of the survey included the EQ-5D-5L assessment.^8^ The EQ-5D-5L is part of a family of standardized surveys developed to provide a simplified measure of health via quality of life. The EQ-5D-5L descriptive system is comprised of 5 dimensions (mobility, self-care, usual activities, pain or discomfort, and anxiety or depression) as well as a visual analog scale (EQ-VAS). The descriptive system consisted of questions with Likert response options ranging 5 levels of severity (no problems, slight problems, moderate problems, severe problems, and unable to perform or extreme problems). The EQ-VAS portion asked participants to rate their own health from a scale of 0 to 100 (with 0 being the worst health imaginable and 100 being the best), providing a quantitative measure of the participant’s perception of their own health. An index score was calculated using the United States EQ-VT v2.0 protocol for preference value sets. Single preference-based indices were then calculated ranging from 1 (best health state) to negative values (health states valued as worse than death), where 0 indicated health state equivalent to death.

The third portion of the survey included administration of the Patient Health Questionnaire-2 (PHQ-2) and Generalized Anxiety Disorder-2 (GAD-2) scales. The PHQ-2 comprised 2 questions which evaluate the core features of depression: having little interest or pleasure in doing things (anhedonia) and feeling down, depressed, or hopeless (depressed mood). Total scores range from 0 to 6, where a score of 3 or above is a diagnostic indicator of depression.^9^ The GAD-2 comprised 2 questions which evaluate the core features of anxiety disorders: feeling nervous, anxious, or on edge and not being able to stop or control worrying. Total scores range from 0 to 6, where a score of 3 or above is a diagnostic indicator of anxiety.^10^ A reported score of 3 or higher on either questionnaire triggered an additional set of questions regarding the well-being of the participant and encouraged the participant to seek help if symptoms became worse.

### Statistical analysis

Summary statistics (i.e., mean, standard deviation (SD), and quartiles) for demographics and clinical characteristics are reported for both cohorts. Continuous variables are summarized as mean ± standard deviation (SD) or mean with 95% confidence intervals (CI) as appropriate and were compared between groups using Student’s t-test or Wilcoxon rank-sum test, depending on distributional assumptions. Categorical variables are summarized as counts and percentages and were compared using chi-square or Fisher’s exact tests as appropriate.

Multivariable linear regression models were constructed to evaluate the association between heart transplant status and summative symptom scores, adjusting for potential confounders including demographic variables (age, sex, race) and clinical factors (pulmonary disease and hypertension). Interaction terms were included to explore whether the effect of heart transplant status on symptom scores differed by the presence of pulmonary disease or hypertension. Interaction between heart transplant status and pulmonary disease was pre-specified based on clinical plausibility and retained in the model. A multivariable regression model was implemented to evaluate the association between OHT status and EQ Index scores, where the model was adjusted for demographic variables, including age, sex, and race/ethnicity. A multivariable regression was implemented to evaluate the association between OHT status and Self-Reported Health, where the model was adjusted for demographic variables, including age, sex, and race, as well as pulmonary disease and hypertension. An odds ratio was calculated with Wald confidence intervals for both the PHQ-2 and GAD-2 assessments.

All statistical tests were two-tailed, and a *p*-value <0.05 was considered statistically significant. Analyses were performed using SPSS.

## Results

A total of 121 patients were included in the study, comprising 66 control patients with CMY and 55 OHTRs who had been infected with COVID-19 at least 3 months prior to the study period **(**Table 1**).** There was a significantly higher LVEF in the OHTR group (62.2% compared to 52.5% in the control group; *p* <0.001). There was a significantly higher proportion of males in the OHTR group (87.27% compared to 72.72% in the control group; *p* = 0.049). The OHTR group had a lower prevalence of pulmonary disease (5.45% compared to 25.76% in the control group; *p* = 0.003) as well as sleep apnea (10.91% compared to 25.76% in the control group; *p* = 0.038). No other significant differences in past medical history were identified.

Between the OHTR group and control group, there was significantly higher administration of remdesivir in the OHTR group (52.73% vs. 9.09%; *p* < 0.001) and convalescent plasma (21.82% vs. 0%; *p* < 0.001), as well as a higher proportion of patients requiring hospitalization (52.73% vs. 18.18%; *p* <0.001). There was a significantly lower administration of nirmatrelvir-ritonavir in the OHTR group (0% vs. 30.3%; *p* < 0.001).

The prevalence of individual Long COVID symptoms between OHTRs and control patients is shown in Table 2. Notably, shortness of breath was less prevalent in OHTRs compared to control patients (10.91% vs. 28.79%; *p* = 0.023), however the difference was not statistically significant after adjustment for multiple comparisons. No other significant differences in symptom prevalence were identified. Symptoms such as post-exertional malaise, difficulty with memory, insomnia, muscle or joint pain, and neuropathy exhibited a high prevalence across both groups but showed no significant variations between the groups.

**Table 2:** Long COVID Symptoms Comparison

**Long-COVID Symptoms - no. (%)**	**Controls (n = 66)**	**Cases (n = 55)**	**Chi-Square**	**Fisher's Exact Test**
Post Exertional Malaise	29 (43.94)	18 (32.73)	0.201	0.262
Shortness of Breath	19 (28.79)	6 (10.91)	0.016	0.023
Loss of Smell	11 (16.67)	5 (9.09)	0.221	0.285
Loss of Taste	8 (12.12)	7 (12.73)	0.92	1
Heartburn	16 (24.24)	9 (16.36)	0.287	0.369
Difficulty with Concentration	22 (33.33)	16 (29.09)	0.617	0.696
Difficulty with Memory	27 (40.91)	17 (30.91)	0.255	
Difficulty Sleeping/Insomnia	21 (31.82)	15 (27.27)	0.586	
Headache	9 (13.64)	8 (14.55)	0.886	
Muscle or Joint Pain	30 (45.45)	24 (43.64)	0.841	
Chest Pain	14 (21.21)	6 (10.91)	0.129	
Palpitations	16 (24.24)	6 (10.91)	0.058	
Chronic Cough	16 (24.24)	9 (16.36)	0.287	
Dizziness/Lightheadedness	17 (25.76)	11 (20)	0.455	
Decreased Appetite	15 (22.73)	15 (27.27)	0.564	
Tinnitus	13 (19.7)	8 (14.55)	0.456	
Nasal Congestion	20 (30.3)	13 (23.64)	0.412	
Nausea/Vomiting	7 (10.61)	3 (5.45)	0.306	0.344
Worsened Vision	19 (28.79)	13 (23.64)	0.522	
Neuropathy	22 (33.33)	14 (25.45)	0.345	

Summary of the prevalence of Long COVID symptoms among cases (n = 55) and controls (n = 66). Statistical analyses (Chi-square and Fisher's Exact tests) were conducted to identify significant differences between the groups.

*No adjustment for multiple comparisons was performed to the *p* values.

A statistically significant interaction between heart transplant status and pulmonary disease was observed for each summative symptom score (*p* = 0.035), EQ index (*p* = 0.017), and self-reported health (*p* = 0.003), indicating that the association between transplant status and these outcomes differed by pulmonary disease status. (Table 3**).** Given the small number of transplant recipients with pulmonary disease (n = 2), these stratified estimates should be interpreted with caution due to potential instability.

**Table 3:** Impact of Heart Transplantation and Pulmonary Disease on Long COVID Measures

	**Summative Symptom Mean Score (95% CI)**	***p*-value**	**EQ Index (95% CI)**	***p*-value**	**Self-Reported Health (95% CI)**	***p*-value**
**No Transplant, No Pulmonary Disease**	4.91 (3.39-6.43)	0.737	0.76 (0.68-0.85)	0.443	76.3 (69.7-82.9)	0.921
**Transplant, No Pulmonary Disease**	4.61 (3.09-6.14)		0.80 (0.72-0.88)		76.0 (69.4-82.6)	
**No Transplant, Pulmonary Disease**	8.23 (6.02-10.44)	0.035	0.46 (0.34-0.58)	0.017	54.1 (44.5-63.7)	0.003
**Transplant, Pulmonary Disease**	2.21 (-3.06-7.47)		0.83 (0.54-1.12)		90.8 (68.0-113.5)	

*Estimates in transplant patients with pulmonary disease should be interpreted cautiously due to small sample size (n = 2).

Among patients with pulmonary disease, the mean symptom score was significantly higher in control patients compared to OHTRs (8.23 [6.02-10.44] vs. 2.21 [-3.06-7.47]; *p* = 0.035) **(**Table 3**).** In contrast, among those without pulmonary disease, there was no significant difference in mean symptom score between the 2 groups (4.91 [3.39-6.43] in the control group vs 4.61 [3.09-6.14] in the OHTR group; *p* = 0.737).

Among patients with pulmonary disease, EQ index scores were significantly lower in control patients compared to OHTRs (0.46 (0.34-0.58) vs. 0.83 (0.54-1.12); *p* = 0.017). In contrast, among those without pulmonary disease, there was no significant difference in EQ index scores between the 2 groups (Table 3).

In the presence of pulmonary disease, self-reported health scores were significantly lower in control patients compared to OHTRs (54.11 (44.50-63.72) vs. 90.75 (67.99-113.51); *p* = 0.003). In contrast, among patients without pulmonary disease, there was no significant difference in self-reported health scores between the 2 groups (Table 3).

There was no significant difference in mental health burden, measured by PHQ-2 and GAD-2, between the OHTR group and control cohort (Table 4).

**Table 4:** Mental Health Burden of COVID on Patients With Heart Transplants

	PHQ-2				GAD-2	
	OR	95% CI	*p*-value	OR	95% CI	*p*-value
**Heart Transplant**	1.19	(0.55, 2.58)	0.667	0.75	(0.35, 1.63)	0.471
**Age**	1.01	(0.98, 1.04)	0.753	1.01	(0.98, 1.04)	0.526
**Sex**						
Female vs Male	1.56	(0.58, 4.18)	0.375	1.29	(0.48, 3.47)	0.608
**Race - compared to White**						
African American	0.63	(0.17, 2.34)	0.384	0.67	(0.19, 2.44)	0.757
Hispanic	1.16	(0.36, 3.71)	0.68	0.71	(0.22, 2.28)	0.805
Other	1.00	(0.37, 2.69)	0.953	0.65	(0.24, 1.74)	0.599
Unknown	1.21	(0.22, 6.82)	0.738	0.96	(0.17, 5.40)	0.754
**Pulmonary Disease**	3.53	(1.17, 10.64)	0.025	3.07	(1.02, 9.23)	0.045

The odds of having PHQ2 > 0 were 3.53 (95% CI: 1.17, 10.64) times higher in individuals with pulmonary disease compared to those without pulmonary disease (p = 0.025). The odds of having GAD2 > 0 were 3.07 (95% CI: 1.02, 9.23) times higher in individuals with pulmonary disease compared to those without pulmonary disease (p = 0.045). *The variable of heart transplantation was not significant. The interaction between heart transplant and pulmonary disease was not significant in both PHQ-2 and GAD-2 analyses

## Discussion

In this study, we examined the impact of Long COVID in an OHTR population. When compared with a control group of non-transplanted cardiomyopathy patients with prior COVID-19, there was no significant difference in the prevalence of Long COVID symptoms in OHTRs. The exception to this finding was a higher incidence of shortness of breath seen in control patients, which was attenuated when controlling for the increased prevalence of pulmonary disease among control patients. There were no significant differences in patient-reported mental health symptoms and quality-of-life metrics between the 2 groups.

The prevalence of Long COVID symptoms is not well defined in the literature. Researchers have estimated the prevalence of symptoms to be between 14.7 and 45 percent, and results are highly variable between studies.^11^, ^12^ Data on the impact of Long COVID in the organ transplant population is further limited with seemingly contradictory findings. Several preliminary studies have found Long COVID prevalence may be low in the heart transplant population.^13^, ^14^ In contrast, the prevalence of Long COVID may be higher among patients who received kidney transplantation,^15^ and kidney transplant recipients on corticosteroid therapy appear to be at increased risk.^25^ Although Long COVID symptoms in this population may be more prevalent, these symptoms have also been described to be attenuated as COVID-19 variants evolve.^19^ These mixed data among SOTRs highlight the need for further research.

To our knowledge, our study is the first to describe the prevalence of Long COVID symptoms among OHT recipients as compared with non-OHT patients with cardiomyopathy. Notably, we did not observe evidence of increased Long COVID symptom burden among OHT recipients compared with cardiomyopathy controls. While our study was not powered to detect a significant decrease in Long COVID symptoms among OHTRs, as we hypothesized a higher prevalence of Long COVID in OHTRs, thus larger studies are needed to more definitively characterize differences in symptom burden.

While the mechanism is poorly understood, it is possible that immunosuppressive medications taken by transplant recipients may be protective against the immune dysregulation that is thought to contribute to Long COVID symptoms. Another hypothesized explanation for the decreased prevalence of Long COVID among transplant recipients may be related to higher rates of antiviral treatment provided to OHTR patients for acute COVID infections. Because transplanted patients on immunosuppressive medications are deemed higher risk for acute COVID-19-related morbidity and mortality, they are more likely to be treated for acute infections as evidenced by our population.

In our sample, OHTR patients were significantly more likely to have received remdesivir and convalescent plasma as compared with non-OHTR patients. In contrast, non-OHTR patients were significantly more likely to have received nirmatrelvir-ritonavir. This is likely in part due to the array of drug-drug interactions that must be considered when administering nirmatrelvir-ritonavir, namely the serious adverse reactions reported with calcineurin inhibitors, which are prescribed to most SOTRs.^20^ Furthermore, a large cohort study found remdesivir is associated with lower mortality rates in immunocompromised patients, including SOTRs.^21^ While remdesivir has been noted to improve mortality from acute COVID infections, it also has a potential role in post-COVID complications, particularly Long COVID symptoms. Though data are limited, remdesivir may have a protective effect against Long COVID in hospitalized patients,^16^ and its use has also shown an association with earlier patient-reported symptom resolution following acute infection in patients previously hospitalized.^23^ However, other studies have shown no difference in patient-reported Long COVID symptoms regardless of treatment with remdesivir.^24^ Given the discrepancies in findings, future studies investigating the potential protective effect of antiviral treatment on development of Long COVID are imperative.

While our study provides several valuable insights into the presence of Long COVID symptoms in the OHTR population, including direct symptom assessment with study participants instead of relying on diagnostic codes from medical chart review, several limitations must be acknowledged. Our relatively small sample size, due to difficulties with patient recruitment, may have limited the statistical power to detect differences in Long COVID symptoms between OHTR and non-OHTR individuals. In particular, subgroup analyses involving pulmonary disease were limited by very small sample sizes and potential contributions of underlying cardiomyopathy limit our ability to discern its impact. Generalizability may also be affected given the significantly higher proportion of males in the OHTR group compared to the control group; however, this demographic difference reflects the higher proportion of males who undergo heart transplantation compared to females.^22^ Additionally, transplant recipients were significantly more likely to have been hospitalized during their acute COVID-19 infection, which may reflect differences in disease severity or treatment exposure. Although multivariable adjustment was performed, residual confounding related to acute illness severity cannot be excluded. Our study also relied on self-reported symptoms, which can be subject to recall bias and individual perception differences. Furthermore, many Long COVID symptoms overlap with baseline symptoms related to transplant, and both groups in this study may have higher baseline exertional limitations, dyspnea, and fatigue independent of SARS-CoV-2; thus, identifying symptoms “worse from baseline” is more difficult to delineate in these populations. This study also did not consider reinfections nor variants of COVID-19 as variables in Long COVID prevalence. However, this study design may offer more granular detail of patient experiences with Long COVID, as prior studies have largely focused on retrospective chart review rather than patient-reported outcomes.^17^, ^18^ Therefore, our study does not demonstrate an increased Long COVID symptom burden among OHT recipients and provides preliminary data to inform future larger investigations.

## Disclosures

None.

## Declaration of Competing Interest

The authors declare that they have no known competing financial interests or personal relationships that could have appeared to influence the work reported in this paper.
